# Determination of 10 Antibiotics and 53 Plant Growth Regulators in Citrus Fruits by QuEChERS Combined with Liquid Chromatography–Quadrupole/Orbitrap Mass Spectrometry

**DOI:** 10.3390/foods15030477

**Published:** 2026-01-30

**Authors:** Yujie Xie, Zhengyi Liu, Mengjie Shi, Xingqiang Wu, Kaixuan Tong, Qiaoying Chang, Chunlin Fan, Hui Chen

**Affiliations:** 1 China Academy of Quality and Inspection & Testing, Beijing 100176, China; xieyj@caiq.org.cn (Y.X.); 17332318156@163.com (M.S.); wuxq@caiq.org.cn (X.W.); tongkx@caiq.org.cn (K.T.); c81618@163.com (Q.C.); caiqfcl@163.com (C.F.); 2 Guizhou Provincial Institute of Food Inspection and Testing, Guiyang 550081, China; 18198288550@163.com

**Keywords:** citrus fruits, antibiotics, plant growth regulators, liquid chromatography–quadrupole/orbitrap mass spectrometry

## Abstract

Citrus fruits are susceptible to ‘Huanglongbing’, leading to widespread antibiotic use during planting. Additionally, to enhance economic efficiency, plant growth regulators (PGRs) are also applied to citrus fruits. To rapidly screen for antibiotics and plant growth regulators in citrus fruits, a method was developed for the simultaneous detection of exogenous contaminants in mandarin, orange, pomelo, and lemon using QuEChERS combined with liquid chromatography–quadrupole/orbitrap mass spectrometry. By comparing the responses or recoveries of compounds under different conditions, the optimal extraction and purification were determined. The method was used to verify the methodological parameters for four citrus fruits. The results showed that the detection limits for 10 antibiotics and 53 plant growth regulators in the four citrus fruits ranged from 1 to 50 μg/kg, and the limits of quantitation ranged from 1 to 80 μg/kg. And the coefficient of determination (*R*^2^) was ≥ 0.99. The recovery of all compounds was between 60% and 120%, and the relative standard deviation (RSD) was less than 20%. The method was applied to the 42 real samples, and a total of nine compounds were detected at concentrations ranging from 0.002 to 0.852 mg/kg. The results demonstrated that the method was simple, sensitive, accurate, and reliable, making it suitable for detecting antibiotics and plant growth regulators in citrus fruits.

## 1. Introduction

Citrus fruits (*Citrus* L.), derived from Rutaceae shrubs, originated in Southeast Asia, such as Yunnan Province of China, Myanmar, etc. It is the fruit with the highest cultivation area and yield in the world. Primary cultivated varieties include mandarin, orange, pomelo, lemon, and kumquat [1,2]. Huanglongbing (HLB) is the most destructive disease of citrus fruit. After infection, fruit yield is reduced, or no harvest occurs, a condition called “citrus cancer” [3]. The pathogen was a Gram-negative bacterium, including the Asian strain (CLas), the American strain (CLam), and the African strain (CLaf). Only CLas was found in China [4,5,6]. Since the 1970s, China has studied the use of antibiotics to prevent and treat HLB. Oxytetracycline, tetracycline, streptomycin, and ampicillin can effectively suppress the pathogen through scion soaking, trunk injection, or foliar spraying [7,8,9]. Among them, oxytetracycline and streptomycin were approved by the U.S. Environmental Protection Agency (EPA) for prevention and treatment [10]. However, the application of these antibiotics raises concerns about pathogen resistance and the risk of cross-species transmission, which may affect the health of animals and humans via horizontal gene transfer (HGT) [11]. At the same time, some countries, such as the European Union and the United States, have established maximum residue limits (MRLs) for antibiotics in citrus fruits [12]. Therefore, the establishment of rapid detection methods for antibiotics in citrus fruits is critical to ensure their quality and safety.

Plant growth regulators (PGRs) are organic compounds, either extracted from microorganisms or chemically synthesized, that mimic the chemical structures and physiological effects of natural plant hormones. PGRs can enhance fruit quality and increase yield and are extensively utilized in modern agricultural production [13,14,15]. The impact of PGRs on plant growth and development is indeed significant, even at low concentrations. For example, spraying early-ripening pomelos with 0.1% brassinosteroids increased yield by 72% [16]. Nevertheless, their excessive application is not without risks, as it can readily induce phytotoxicity, including fruit deformity and a decline in tree vigor [17], posing a serious threat to fruit marketability and the sustainable management of orchards. Therefore, the establishment of rapid detection methods for PGR residues in citrus fruits is essential, as it will be a crucial step toward promoting the high-quality and sustainable development of the citrus industry.

According to the literature, detection methods for antibiotics have been established for animal-derived foods [18,19]. However, some literature reports on the detection technology for antibiotic residues in plant-derived foods [20,21], indicating that their presence is becoming increasingly common. At the same time, because of the higher infection rate of HLB in citrus fruits, antibiotics were used to prevent and treat HLB, and antibiotic residues will appear in citrus fruits. On the contrary, the detection methods for PGRs in plant-derived foods are more mature than those for antibiotics [22,23]. Although PGR methods are numerous, the detection samples are mainly singular, and the detection instruments are mostly LC-MS/MS. For example, Su et al. established a method for pesticides and PGRs in cherries by LC-MS/MS and used the technology to detect compounds in cherry cultivation in the main areas [24]. Yue et al. used QuEChERS combined with LC-MS/MS to detect 19 PGRs and fungicides in Radix Ophiopogonis, and the limit of quantification (LOQ) was 0.16–5.61 μg/kg [25]. The above detection methods are sensitive; they have the disadvantages of limited target compounds and incomplete detection, making it challenging to meet the needs of high-throughput detection. In recent years, high-resolution mass spectrometry (HRMS) technology has become increasingly popular and is widely used to detect chemical hazards, such as pesticides and veterinary drugs. This technology not only detects various compounds but also offers additional advantages for screening unknown compounds [26]. To realize the simultaneous and rapid detection of antibiotics and PGRs in citrus fruits, based on liquid chromatography–quadrupole/electrostatic field orbitrap mass spectrometry (LC-Q-orbitrap/MS), a method for the simultaneous determination of 10 antibiotics and 53 plant growth regulators in citrus (including mandarin, orange, grapefruit and lemon) was established by optimizing the preparation conditions and successfully applied to the analysis of actual samples. The purpose of this study is to provide robust technical support for the monitoring of exogenous pollutants in citrus to promote the high-quality, sustainable development of the industry.

## 2. Materials and Methods

### 2.1. Instrumentation

The ultrahigh-performance liquid chromatography–quadrupole/orbitrap mass spectrometry consisted of the Ultimate 3000 UHPLC system (Dionex Corporation, Sunnyvale, CA, USA) in conjunction with Q-Orbitrap mass spectrometer from Thermo Fisher Scientific (Bremen, Germany). AH-30 Fully Automatic Homogenizer and Auto EVA 80 Fully Automatic Parallel Nitrogen Blowing Concentrator were obtained from Raykol Instrument Co., Ltd. (Xiamen, China); PL602-L electronic balance was purchased from Mettler-Toledo (Zurich, Switzerland); SR-2DS oscillator (Taitec, Saitama, Japan), a KDC-40 low-speed centrifuge (Zonkia, Hefei, China), ultrasonic cleaner (Kunshan, China), and a Milli-Q ultrapure water machine (Milford, MA, USA) were also purchased.

### 2.2. Materials and Reagents

The standards of 63 compounds (purity grade, >92%) were from Alta Company (Alta, Tianjin, China); ceramic homogenizer was from Agilent Technologies (Santa Clara, CA, USA); formic acid, ammonium acetate, methanol, and acetonitrile (all LC-MS grade) were obtained from Fisher Scientific, Inc. (Fair Lawn, NJ, USA); and acetic acid, NaCl, anhydrous Na_2_SO_4_, ethylenediaminetetraacetic acid disodium salt, disodium hydrogen phosphate, and citric acid (analytical grade forms) were obtained from Beijing Chemical Plant (Beijing, China). The appropriate sorbent of primary secondary amine (PSA) and octadecylsilane (C_18_) were obtained from Tianjin Agela Technology (Tianjin, China).

### 2.3. Standard Solution Preparation

A total of 10 antibiotics and 53 plant growth regulators were transferred into a 10 mL brown volumetric flask, and the volume was adjusted to scale with methanol. After ultrasound, a mixed standard working solution of 10 mg/L was obtained and stored in a brown storage liquid bottle. The solution was stored at 4 °C in the dark.

### 2.4. Sample Collection

A total of 42 samples (14 mandarin, 10 orange, 8 pomelo, and 10 lemon) were collected from fruit stores and supermarkets in Beijing, and all weighed more than 500 g. The sample was ground using a homogenizer after removing the fruit stalk and stored at −20 °C.

### 2.5. Instrument Parameters

Chromatographic separation was achieved under the following chromatographic conditions: equipped with reversed-phase chromatography column (Accucore aQ 150 × 2.1 mm, 2.6 μm); column temperature: 40 °C; injection volume: 5 µL; mobile phases A and B were 5 mM ammonium acetate −0.1% formic acid–water and 0.1% formic acid–methanol, respectively; gradient elution program, 0 min to 3 min; the mobile phase B was 1% to 30%; 3 min to 6 min, the mobile phase B was 30% to 40%; from 6 min to 9 min, the mobile phase B was 40%; from 9 min to 15 min, the mobile phase B was 40% to 60%; from 15 min to 19 min, the mobile phase B was 60% to 90%; from 19 min to 23 min, the mobile phase B was 90%; from 23 min to 23.01 min, the mobile phase B was 90% to 1% (run after 4 min); flow rate was 0.4 mL/min.

Heated Electrospray Ionization (HESI) was used on the Q-Orbitrap in negative and positive ionization modes. The conditions for electrospray ionization were set as follows: scan mode: full scan/data dependent secondary scan (Full MS/dd-MS^2^); Full MS scan range: 80–1100 *m/z*; resolution: 70,000 Full Width at Half Maximum (FHWM), Full MS; and MS^2^ was 17,500 FHWM; maximum injection time was 200 ms and 60 ms for Full MS and MS^2^; automatic gain control of Full MS and MS^2^ was 1 × 10^6^ and 2 × 10^5^, respectively; loop count and multiplex count were 1; Isolation width: 2.0 *m*/*z*; under fill ratio: 1%; stepped normalized collision energy: 20, 40, and 60; apex trigger: 2–6 s; and dynamic exclusion: 8 s. The information on MS is in Table 1.

### 2.6. Sample Preparation

Accurately weigh 10 g (±0.01 g) of the sample into a 100 mL centrifuge tube, add 5 mL of Na_2_EDTA-Mcllvaine buffer solution (37.2 g ethylenediaminetetraacetic acid disodium salt, 27.5 g disodium hydrogen phosphate and 12.0 g citric acid, water volume to 1000 mL, and adjust pH to 4.0) and 20 mL of 3% acetic acid acetonitrile, and homogenize for 1 min. Then, the ceramic homogenizer, 4 g anhydrous Na_2_SO_4_, and 1 g NaCl were added, shaken for 20 min, and centrifuged at 4500 r/min for 5 min. A total of 8 mL of the supernatant was added to a centrifuge tube (containing 100 mg C_18_ and 200 mg anhydrous MgSO_4_), shaken for 10 min, and centrifuged at 4500 r/min for 5 min. After which, 4 mL of the supernatant was pipetted into a tube, evaporated to dryness in a 40 °C water bath under a gentle stream of nitrogen, then dissolved in 1 mL of acetonitrile/water (3:2, *v*/*v*), and filtered through a 0.22 μm filter for LC-Q-Orbitrap/MS analysis.

### 2.7. Method Validation

The method was validated across four citrus fruits by evaluating matrix effect, limits of detection (LODs), limits of quantification (LOQs), accuracy, and precision. Matrix effects were evaluated by comparing the slope of the matrix-matched calibration curve with the solvent calibration curve. LOD and LOQ were the signal-to-noise ratios (S/N) ≥ 3 and the signal-to-noise ratios (S/N) ≥ 10 at the lowest addition level of the compound, respectively. Meanwhile, the recovery ranged from 60% to 120%, and the relative standard deviation (RSD) was <20% for LOQ. The accuracy and precision of the compounds were verified at 1× LOQ, 2× LOQ, and 10× LOQ, with six replicates at each spiked level.

Thermo Fisher Scientific ^TM^ Tracefinder ^TM^ (version 4.1, Waltham, MA, USA) software was used to analyze the data based on the self-built database. The data results were analyzed using Excel (Version 2019) software, and an analysis of graphs was drawn using Origin 2024 software.

## 3. Results and Discussion

### 3.1. Optimization of Extraction Solvent Acidity and Volume

**Figure 1:**
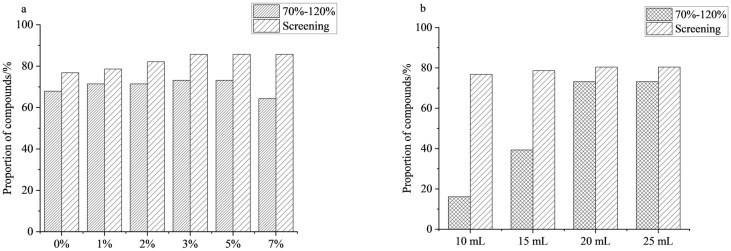
Effect of extraction solvents on the proportion of compounds: (**a**) acidity of extraction solvents; (**b**) volume of extraction solvents (*n* = 3).

Acetonitrile was employed as the extraction solvent due to its strong polarity, which enables efficient extraction of compounds while significantly reducing co-soluble impurities [27,28]. According to the literature, most PGRs exhibit higher extraction efficiency under acidic conditions, with substantially greater stability than under neutral conditions [29]. Therefore, using mandarin as a matrix, this study compared the extraction recoveries of the compounds using acetonitrile with different concentrations of acetic acid (0%, 1%, 2%, 3%, 5%, 7%). As shown in Figure 1a, with increasing acetic acid concentration, the number of compounds with recoveries in the 70–120% range increased first, then decreased. Optimal recovery was observed at acetic acid concentrations of 3–5%. Under these conditions, 73.2% of the compounds met the recovery criteria, and 85.7% were successfully detected. When the acetic acid content was further increased to 7%, the proportion of compounds satisfying the recovery criteria decreased to 64.3%, while the detection rate remained unchanged. In summary, 3% and 5% acetic acid in acetonitrile provided the best extraction performance. Between them, 3% acetic acid in acetonitrile was ultimately selected as the optimal extraction condition for subsequent analysis.

To further enhance extraction efficiency, the effect of extraction solvent volume (10, 15, 20, and 25 mL) on compound recovery was investigated. The results are shown in Figure 1b; the number of recoveries for the compounds increased with increasing extraction volume. When the extraction volume was 10 mL, only nine compounds met the recovery criteria, yielding a qualified rate of 16.1%, indicating that insufficient solvent volume hampered complete extraction. As the extraction volume increased, the number of compounds meeting the recovery criteria gradually increased. When the volume increased to 20 mL, the number of qualified compounds reached a maximum, 4.6 times that at 10 mL. However, when the volume was further expanded to 25 mL, the number of qualified compounds remained the same as at 20 mL, and the RSD increased, indicating reduced repeatability. Based on the experimental results, 20 mL was finally determined to be the optimal extraction solvent volume.

### 3.2. Optimization of the Type of Salt

**Figure 2:**
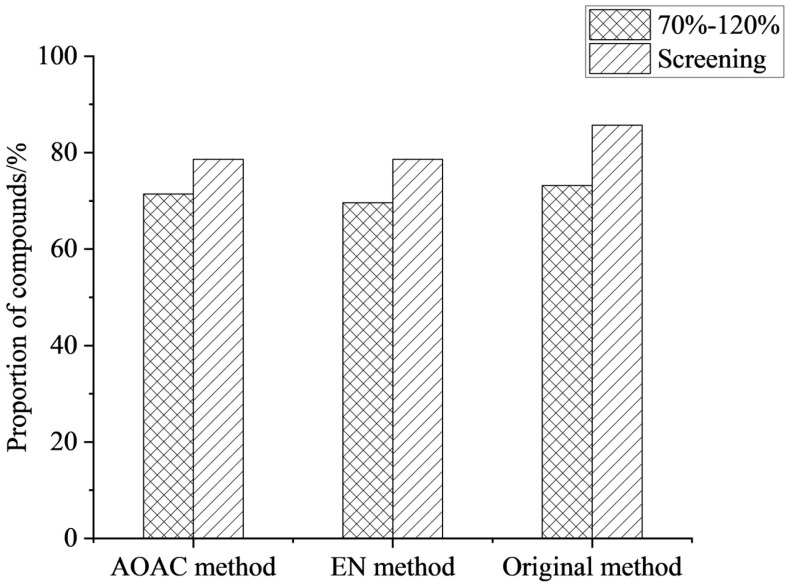
Effect of different types of salts on the proportion of compounds (*n* = 3).

During extraction, the choice of salt is crucial for improving compound recovery. The core mechanism was to induce the separation of the miscible acetonitrile and aqueous phases by significantly increasing the ionic strength of the aqueous phase [30]. The addition of salt to the extraction solution substantially alters the distribution behavior of the compounds between the two phases, driving their transfer from the aqueous phase to the organic extraction phase, thereby achieving efficient extraction [31]. In this work, the extraction effects of the original method (4 g Na_2_SO_4_ + 1 g NaCl), EN (4 g Na_2_SO_4_ + 1 g NaCl + 0.5 g disodium hydrogen citrate + 1 g sodium citrate), and AOAC (6 g Na_2_SO_4_ + 1.5 g NaAc) were compared. The results showed that the three salting agents had little effect on the recoveries of the compounds, but the compound detection rate of the original method was 85.7%, slightly higher than that of the other two methods (see Figure 2). Therefore, the original method (4 g Na_2_SO_4_ + 1 g NaCl) was selected as the extraction salts for this work.

### 3.3. Optimization of the Adsorbent

For QuEChERS, the commonly used purification absorbents include PSA, C_18,_ and MgSO_4_. PSA removed numerous impurities, such as organic acids, sugars, and pigments, and adsorbed compounds containing carboxyl groups. C_18_ exhibits hydrophobic properties and can be used to remove non-polar impurities; it effectively removes interfering substances such as fatty acids and fat-soluble pigments. MgSO_4_ removed water from the matrix to promote the separation of acetonitrile from the aqueous phase [32,33]. The presence of endogenous components, including abundant natural pigments, sugars, and organic acids in citrus fruits, can interfere with the detection of compounds. To improve quantitative accuracy and avoid instrument pollution, the three purification absorbents of MgSO_4_, PSA, and C_18_ were optimized in this work. The factors and levels were designed for MgSO_4_ (0 mg, 200 mg, 400 mg, 600 mg), PSA (0 mg, 100 mg, 200 mg, 300 mg), and C_18_ (0 mg, 100 mg, 200 mg, 300 mg) by an orthogonal experimental design table (L_16_(4^3^)), with 16 runs and three experimental factors at four levels [34]. Experiments were conducted to determine each factor’s primary and secondary effects on recovery based on the proportion of compounds in each treatment group. The results were analyzed using analysis of variance (ANOVA) to determine the best purification absorbents. The results of the orthogonal test of purification absorbents are shown in Table 2, and the ANOVA results are presented in Table 3.

According to the analysis of orthogonal experiment results, the higher R value indicated greater importance of the factor in the experiment. The results in Table 2 showed that the three factors were significant for the compounds, with A > C > B, and the K values were larger when factor A was at level 1, and factors B and C were both at level 3. Simultaneously, the ANOVA results in Table 3 indicated that factors A and C significantly affect the recoveries of the compounds (*p* < 0.05), while factor B has no significant effect (*p* > 0.05). However, as the amount of factor B increases, the ability to eliminate interference also increases. Conversely, excessive amounts could lead to reduced recoveries due to adsorption of the compounds. Therefore, in combination with the results of Table 2 and Table 3, this work ultimately selected 200 mg MgSO_4_ and 100 mg C_18_ as the purification filler.

### 3.4. Optimization of the Volume of Evaporation

**Figure 3:**
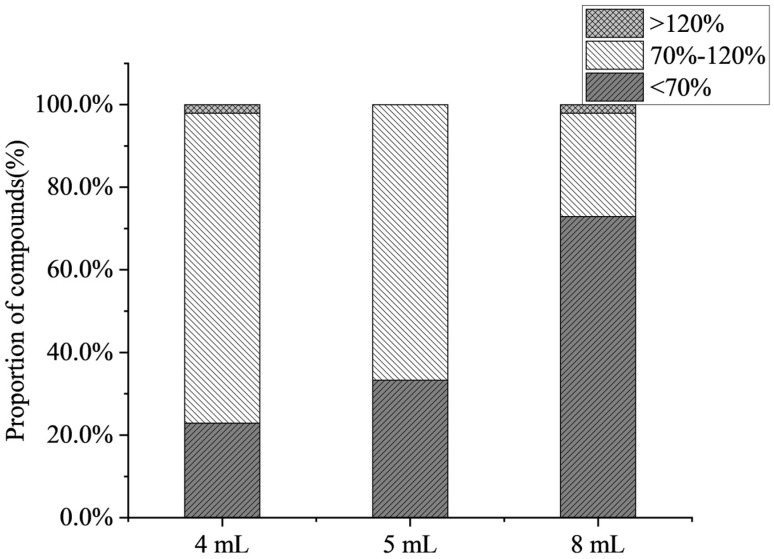
Effect of the volume of evaporation on the proportion of compounds (*n* = 3).

The effects of different volumes of evaporation (4 mL, 5 mL, 8 mL) on the recoveries of compounds were investigated in this work. Figure 3 shows that the average recovery decreased with an increase in the volume of evaporation, and some compounds (such as 6-benzylaminopurine and chlorphonium) exhibited a declining recovery trend. When the volume of evaporation was 8 mL, only 25% of the target compound recovery was accepted. The results indicate that as the volume of evaporation increased, the matrix effect increased, and antibiotics and PGRs were mostly inhibited in citrus fruits. At the volume of evaporation of 4 mL, the highest number of compounds was detected, achieving a detection rate of 91.1%. Therefore, the volume of evaporation of 4 mL was selected.

### 3.5. Matrix Effect

Matrix effect (ME) occurs when interfering substances in the sample enter the ion source with the target compound, altering the compound’s ionization efficiency and thereby affecting the method’s accuracy and sensitivity [35]. The calculation formula of matrix effect was used: matrix effect (ME, %) = [(*K*_1_ − *K*_2_)/*K*_2_] × 100%, where K1 and K2 are the slopes of the matrix-matched standard curve and the slope of the solvent standard curve, respectively. The results showed that |ME| was lower than 20%, between 20% and 50%, or higher than 50%, indicating weak, medium, and strong matrix effects, respectively. Matrix effect is displayed in Table 4. Overall, the primary effects observed were weak matrix effects and medium matrix effects in the four citrus fruits, accounting for 74.6%, 66.7%, 63.5%, and 57.1% of the total, respectively. Except for Oleandomycin and Sulfadimethoxine, all antibiotics showed strong matrix effects, accounting for 80.0%; the PGRs mainly showed weak and medium matrix effects, with proportions of 84.9%, 75.5%, 71.7%, and 64.2% for the four citrus fruits, respectively.

### 3.6. Linear Range, LOD, and LOQ

The mixed standard working solution was added to the blank sample, the sample was extracted and purified according to 2.6, and a matrix-matched standard curve was established. LC-Q-Orbitrap/MS was used to detect the compounds. The results showed that the determination coefficients (*R*^2^) of the compounds in four citrus fruits were all greater than 0.99 (see Table 4). In addition to the compounds listed in Table 4, six compounds were not included due to poor *R*^2^ values in pomelo and lemon. The LODs in four citrus fruits were 1–50 µg/kg, with the LOQs being 2–80 µg/kg in mandarin and orange and 2–50 µg/kg in pomelo and lemon for 10 antibiotics. For 53 plant growth regulators, the LODs were 1–20 µg/kg in mandarin and pomelo and 2–50 µg/kg in orange and lemon, while the LOQs in four citrus fruits were 1–20 µg/kg, 1–50 µg/kg, 1–50 µg/kg, and 1–80 µg/kg, respectively. The proportions of the target compound with LOQ ≤ 5 µg/kg were 66.7%, 61.9%, 68.3%, and 63.5%, respectively.

### 3.7. Recovery and Precision

Results shown in Table 5 indicate that the recovery of all compounds in mandarin, orange, pomelo, and lemon was 61.4–119.9%, 62.6–119.9%, 61.4–119.8%, and 62.6–119.6%, respectively, and the relative standard deviations (RSDs) of the four citrus fruits were lower than 20%. For antibiotics, the recovery rates of four citrus fruits were 61.8–118.6%, 70.4–119.6%, 63.3–118.0%, and 71.2–116.5%, respectively. The recoveries of PGRs in four citrus fruits were 61.4–119.9%, 62.6–119.9%, 61.4–119.8%, and 62.6–119.6%, respectively. The result indicated that the method had good accuracy and precision and could accurately quantify all compounds in four citrus fruits simultaneously.

### 3.8. Analysis of Real Samples

The established method was applied to 14 batches of mandarin, 10 batches of orange, 8 batches of pomelo, and 10 batches of lemon. The results (Table 6) revealed that nine compounds had an amount of 35 times, including 1 antibiotic amount of 12 times and 8 PGR amounts of 23 times. Antibiotic was detected in lemon, and the concentration range was 0.059–0.120 mg/kg. The PGRs were detected in three other citrus fruits; among them, there were five compounds in mandarin, six compounds in orange, and two compounds in pomelo, with the concentration ranges being 0.004–0.165 mg/kg, 0.002–0.084 mg/kg, and 0.006–0.852 mg/kg, respectively. Based on the National Food Safety Standard—Maximum residue limits of pesticides in food (GB 2763-2021), the result of the compounds did not exceed the limit value [36].

## 4. Conclusions

In citrus cultivation, the widespread use of antibiotics and PGRs has led to detectable residues of these compounds in citrus fruits due to the prevalence of infections. In order to achieve simultaneous detection of antibiotics and PGRs in citrus fruits, a method for the detection of 10 antibiotics and 53 plant growth regulators in citrus fruits by QuEChERS combined with LC-Q-Orbitrap/MS was established by optimizing the pretreatment conditions in this work. Overall, 63 compounds passed the validation with satisfactory recoveries and exhibited a good sensitivity in four citrus fruits through the validation of methodological parameters such as matrix effect, limit of determination, limit of quantitation, determination coefficients, and recovery. The results showed that the determination coefficients of all compounds were good (*R*^2^ ≥ 0.99), and the recoveries were between 60% and 120%. RSD was <20%. The LOQs in four citrus fruits were 2–80 µg/kg for 10 antibiotics and 1–80 µg/kg for PGRs. This method analyzed 42 batches of citrus fruits, and a total of one antibiotic and eight PGRs were detected. The result indicated that the method is characterized by rapidity, simplicity, and sensitivity. It not only rapidly screens antibiotics and PGRs in citrus fruits but also provides support for other citrus fruits.

## Figures and Tables

**Table 1:** The information on 10 antibiotics and 53 plant growth regulators.

No.	Compounds	Types	Polarity	Formula	RT (min)	Quantitative Ion	Qualitative Ion
1	4-Epi-Chlortetracycline	Antibiotics	Positive	C_22_H_23_ClN_2_O_8_	4.92	479.1200	444.08514, 462.09567
2	4-Epi-Oxytetracycline	Antibiotics	Positive	C_22_H_24_N_2_O_9_	4.13	461.15738	426.11850, 444.12939
3	Chlortetracycline	Antibiotics	Positive	C_22_H_23_ClN_2_O_8_	5.63	479.11951	444.08533, 462.09546
4	Doxycycline	Antibiotics	Positive	C_22_H_24_N_2_O_8_	7.09	445.15988	428.13455, 321.07605
5	Methacycline	Antibiotics	Positive	C_22_H_22_N_2_O_8_	6.50	443.14493	426.11896, 201.05487
6	Minocycline	Antibiotics	Positive	C_23_H_27_N_3_O_7_	4.19	458.19293	441.16592, 283.08405
7	Oleandomycin	Antibiotics	Positive	C_35_H_61_NO_12_	10.33	688.42651	158.11780, 544.34888
8	Oxytetracycline	Antibiotics	Positive	C_22_H_24_N_2_O_9_	4.31	461.15738	426.11850, 201.05478
9	Sulfadimethoxine	Antibiotics	Positive	C_12_H_14_N_4_O_4_S	5.99	311.08084	156.07695, 108.04449
10	Tetracycline	Antibiotics	Positive	C_22_H_24_N_2_O_8_	4.29	445.16125	410.12375, 154.04991
11	1,3-Diphenyl urea	PGRs	Positive	C_13_H_12_N_2_O	11.05	213.10214	94.06525, 120.04469
12	1-Naphthyl acetamide	PGRs	Positive	C_12_H_11_NO	7.55	186.09128	141.07004, 169.06496
13	2,4,5-T	PGRs	Negative	C_8_H_5_Cl_3_O_3_	13.43	252.92311	194.91769, 158.94096
14	2,4-D	PGRs	Negative	C_8_H_6_Cl_2_O_3_	9.76	218.96204	160.95665, 124.97991
15	2-Naphthyloxyacetic acid	PGRs	Negative	C_12_H_10_O_3_	9.50	201.05565	143.05017, 157.06573
16	2-Pyridylpropanol	PGRs	Positive	C_8_H_11_NO	0.86	138.09149	120.08089, 92.04930
17	3-Indolyl	PGRs	Positive	C_10_H_9_NO_2_	5.75	176.07077	130.06534, 158.06036
18	4-Bromophenoxyacetic acid	PGRs	Negative	C_8_H_7_BrO_3_	7.65	228.95053	170.94514, 78.91891
19	4-Chlorophenoxyacetic acid	PGRs	Negative	C_8_H_7_ClO_3_	6.99	185.00107	111.00077, 91.01884
20	4-Fluorophenoxyacetic acid	PGRs	Negative	C_8_H_7_FO_3_	5.01	169.03055	111.02522, 95.03033
21	4-Iodophenoxyacetic acid	PGRs	Negative	C_8_H_7_IO_3_	9.07	276.93661	126.90501, 218.93126
22	4-Nitrophenol	PGRs	Negative	C_6_H_5_NO_3_	5.37	138.01953	108.02173, 92.02676
23	5-Nitroguaiacolate	PGRs	Negative	C_7_H_6_NO_4_	5.68	168.03015	153.00670, 123.00882
24	6-Benzylaminopurine	PGRs	Positive	C_12_H_11_N_5_	6.95	226.10887	91.05434, 148.06206
25	6-Isopentenyl aminopurine	PGRs	Positive	C_10_H_13_N_5_	6.83	204.12343	136.06206, 148.06200
26	Atrazine	PGRs	Positive	C_8_H_14_ClN_5_	11.99	216.10185	174.05428, 96.05568
27	Avermectin B1a	PGRs	Positive	C_48_H_72_O_14_	20.28	890.5260	305.21121, 95.04922
28	Benzanilide	PGRs	Positive	C_13_H_11_NO	9.48	198.09135	105.03364, 120.04456
29	Butralin	PGRs	Positive	C_14_H_21_N_3_O_4_	19.85	296.16071	240.09789, 222.08740
30	Carbaryl	PGRs	Positive	C_12_H_11_NO_2_	10.09	202.08595	145.06499, 117.06993
31	Chlormequat	PGRs	Positive	C_5_H_13_ClN	0.91	122.07322	58.06538, 62.99980
32	Chlorphonium	PGRs	Positive	C_19_H_32_Cl_2_P	16.03	361.16312	158.97644, 173.14552
33	Cloprop	PGRs	Negative	C_9_H_9_ClO_3_	9.50	199.01671	126.99564, 71.01391
34	Cyclanilide	PGRs	Positive	C_11_H_9_Cl_2_NO_3_	16.23	274.00311	113.02334, 161.98734
35	Cycloheximide	PGRs	Positive	C_15_H_23_NO_4_	6.59	282.17004	264.15939, 246.14906
36	Daminozide	PGRs	Positive	C_6_H_12_N_2_O_3_	0.99	161.09174	143.08162, 115.08686
37	Dichlorprop	PGRs	Negative	C_9_H_8_Cl_2_O_3_	13.56	232.97765	160.95663, 124.97989
38	Diethyl Aminoethyl Hexanoate	PGRs	Positive	C_12_H_25_NO_2_	5.66	216.19623	143.10687, 100.11216
39	Diniconazole	PGRs	Positive	C_15_H_17_Cl_2_N_3_O	18.53	326.08215	70.04006, 158.97633
40	Ethychlozate	PGRs	Positive	C_11_H_11_ClN_2_O_2_	14.35	239.05890	165.02156, 193.01648
41	Ethyl 1-naphthylacetate	PGRs	Positive	C_14_H_14_O_2_	16.95	215.10715	141.07001
42	Flurprimidol	PGRs	Positive	C_15_H_15_F_3_N_2_O_2_	16.27	313.11560	270.06094, 269.05331
43	Forchlorfenuron	PGRs	Positive	C_12_H_10_ClN_3_O	12.93	248.05859	129.02147, 155.00073
44	Gibberellic	PGRs	Negative	C_19_H_22_O_6_	5.16	345.13437	143.08655, 221.13356
45	Guayule	PGRs	Positive	C_12_H_17_Cl_2_NO	7.51	262.07626	100.11218, 58.06541
46	Inabenfide	PGRs	Positive	C_19_H_15_ClN_2_O_2_	15.09	339.08960	321.07904, 214.04233
47	Kinetin	PGRs	Positive	C_10_H_9_N_5_O	5.00	216.08723	81.03356, 216.08817
48	Mefluidide	PGRs	Positive	C_11_H_13_F_3_N_2_O_3_S	8.93	311.06705	135.09177, 121.07610
49	Mepiquat	PGRs	Positive	C_7_H_16_N	0.98	114.12785	98.09644, 58.06533
50	Paclobutrazol	PGRs	Positive	C_15_H_20_ClN_3_O	15.94	294.13669	70.04009, 125.01538
51	Phenazine-1-carboxylic acid	PGRs	Positive	C_13_H_8_N_2_O_2_	11.35	225.06573	207.05545, 179.06055
52	Prohexadione	PGRs	Negative	C_10_H_12_O_5_	6.06	211.06116	167.07130, 123.08155
53	Prohydrojasmon	PGRs	Positive	C_15_H_26_O_3_	19.52	255.19545	153.12752, 135.11716
54	Pyraflufen-ethyl	PGRs	Positive	C_15_H_13_Cl_2_F_3_N_2_O_4_	18.13	413.02795	338.99112, 260.99924
55	Pyribenzoxim	PGRs	Positive	C_32_H_27_N_5_O_8_	19.22	610.19324	180.08098, 413.10965
56	Simazine	PGRs	Positive	C_7_H_12_ClN_5_	8.55	202.08569	132.03246, 104.00108
57	Thiabendazole	PGRs	Positive	C_10_H_7_N_3_S	4.84	202.04349	175.03261, 131.06047
58	Thidiazuron	PGRs	Positive	C_9_H_8_N_4_OS	8.25	221.04918	102.01215, 94.06523
59	Triadimefon	PGRs	Positive	C_14_H_16_ClN_3_O_2_	16.33	294.10098	197.07289, 141.01027
60	Triapenthenol	PGRs	Positive	C_15_H_25_N_3_O	17.58	264.20804	70.04008, 95.08558
61	Tribufos	PGRs	Positive	C_12_H_27_OPS_3_	20.23	315.10269	112.92802, 168.99068
62	Uniconazole	PGRs	Positive	C_15_H_18_ClN_3_O	17.46	292.12125	70.04005, 125.01534
63	Zeatin	PGRs	Positive	C_10_H_13_N_5_O	3.62	220.11821	136.06197, 148.06180

**Table 2:** Purification results of the L_16_(4^3^) sample’s orthogonal test.

Trial No.	Factors			Average Recovery (%)
	A ^a^	B ^b^	C ^c^	
1	1	1	1	78.7
2	1	2	3	83.0
3	1	3	4	76.6
4	1	4	2	87.2
5	2	1	2	68.1
6	2	2	4	70.2
7	2	3	3	70.2
8	2	4	1	57.4
9	3	1	3	74.5
10	3	2	1	70.2
11	3	3	2	78.7
12	3	4	4	72.3
13	4	1	4	63.8
14	4	2	2	70.2
15	4	3	1	61.7
16	4	4	3	70.2
K1	81.4	71.3	67.0	
K2	66.5	73.4	76.1	
K3	73.9	71.8	74.5	
K4	66.5	71.8	70.7	
R	14.9	2.1	9.0	
Optimum level	1	2	2	

^a^ The amount of PSA: level 1, 0 mg; level 2, 100 mg; level 3, 200 mg; level 4, 300 mg. ^b^ The amount of C_18_: level 1, 0 mg; level 2, 100 mg; level 3, 200 mg; level 4, 300 mg. ^c^ The amount of MgSO_4_: level 1, 0 mg; level 2, 200 mg; level 3, 400 mg; level 4, 600 mg.

**Table 3:** Analysis of ANOVA experiment results.

Source	Sum of Squares	*df*	Mean Square	*F*	*p*
Intercept	18,360.25	1	18,360.25	7107.194	0.000 **
A	134.75	3	44.917	17.387	0.002 **
B	2.25	3	0.75	0.29	0.831
C	43.25	3	14.417	5.581	0.036 *
Residual	15.5	6	2.583		
R^2^ = 0.921					

* *p* < 0.05; ** *p* < 0.01.

**Table 4:** Determination coefficients, the limits of detection (LODs), limits of quantification (LOQs), and matrix effect (ME) of 10 antibiotics and 53 PGRs in four citrus fruits.

No.	Compounds	Mandarin				Orange				Pomelo				Lemon			
		LOD	LOQ	*R* ^2^	ME (%)	LOD	LOQ	*R* ^2^	ME (%)	LOD	LOQ	*R* ^2^	ME (%)	LOD	LOQ	*R* ^2^	ME (%)
		(μg/kg)	(μg/kg)			(μg/kg)	(μg/kg)			(μg/kg)	μg/kg)			(μg/kg)	(μg/kg)		
Antibiotics (10)																	
1	4-Epi-Chlortetracycline	20	50	0.9965	105.12	50	50	0.9927	410.50	20	50	0.9970	243.79	5	10	0.9973	993.08
2	4-Epi-Oxytetracycline	20	50	0.9920	1153.58	50	80	0.9977	2951.26	50	50	0.9933	2940.94	20	50	0.9985	1728.00
3	Chlortetracycline	20	50	0.9943	73.96	20	20	0.9904	632.58	20	50	0.9947	390.03	50	50	0.9921	663.23
4	Doxycycline	20	50	0.9906	92.13	50	80	0.9923	251.61	50	50	0.9987	394.75	50	50	0.9923	542.80
5	Methacycline	50	80	0.9963	803.95	20	20	0.9924	360.58	20	50	0.9986	416.70	50	50	0.9966	531.18
6	Minocycline	20	50	0.9932	957.91	20	20	0.9900	532.27	50	50	0.9973	530.99	50	50	0.9947	713.89
7	Oleandomycin	5	5	0.9998	−31.72	5	5	0.9990	−49.01	2	5	0.9931	−33.42	5	10	0.9962	−35.56
8	Oxytetracycline	20	20	0.9920	2851.74	20	80	0.9904	1382.73	20	50	0.9929	1119.61	20	50	0.9985	1838.54
9	Sulfadimethoxine	1	5	0.9947	3.95	1	2	0.9945	−34.06	1	2	0.9999	5.77	2	10	0.9930	−32.37
10	Tetracycline	2	2	0.9929	1670.91	2	2	0.9950	616.75	2	5	0.9938	655.73	1	2	0.9906	906.85
Plant Growth Regulators (53)																	
1	1,3-Diphenyl urea	1	1	0.9956	−5.00	1	2	0.9967	−10.85	1	1	0.9990	−32.78	1	1	0.9999	−36.00
2	1-Naphthyl acetamide	1	1	0.9993	−37.33	1	1	0.9979	−38.87	1	1	0.9982	−49.43	1	2	0.9997	−40.02
3	2,4,5-T	10	10	0.9989	68.83	5	10	0.9988	74.54	2	5	0.9998	109.29	5	5	0.9998	85.96
4	2,4-D	1	2	0.9920	80.11	10	10	0.9997	35.25	10	10	0.9969	20.13	5	5	0.9969	64.75
5	2-Naphthyloxyacetic acid	10	10	0.9995	1.64	10	10	0.9985	16.05	10	10	0.9932	12.17	5	5	0.9932	60.27
6	2-Pyridylpropanol	1	1	0.9944	−74.19	1	1	0.9978	−79.54	1	1	0.9905	−72.23	1	1	0.9902	−75.88
7	3-Indolyl	1	2	0.9993	13.98	1	2	0.9995	−21.23	1	5	0.9981	−27.60	1	2	0.9999	−17.65
8	4-Bromophenoxyacetic acid	10	10	0.9943	2.19	10	10	0.9974	−8.35	10	20	0.9938	−26.15	10	10	0.9938	−25.31
9	4-Chlorophenoxyacetic acid	10	10	0.9998	23.76	5	10	0.9985	18.51	5	5	0.9977	−0.56	5	10	0.9977	22.15
10	4-Fluorophenoxyacetic acid	10	10	0.9993	9.09	5	10	0.9990	6.74	5	5	0.9997	−2.28	10	10	0.9997	−22.33
11	4-Iodophenoxyacetic acid	10	10	0.9990	19.23	5	10	0.9958	19.46	10	10	0.9972	22.73	5	5	0.9972	17.64
12	4-Nitrophenol	1	2	0.9957	−35.04	2	2	0.9977	−32.06	2	2	0.9986	−32.48	2	2	0.9986	−32.81
13	5-Nitroguaiacolate	10	20	0.9916	−11.01	10	10	0.9990	−11.11	10	10	0.994	−23.59	10	10	0.9940	−32.85
14	6-Benzylaminopurine	1	2	0.9985	−35.98	1	1	0.9981	−56.52	1	2	0.9987	−51.83	1	1	0.9994	−65.39
15	6-Isopentenyl aminopurine	1	2	0.9965	−30.64	1	1	0.9983	−51.15	1	2	0.9997	−39.74	1	2	0.9992	−53.05
16	Atrazine	1	1	0.9997	−14.41	1	1	0.9956	−43.43	1	2	0.9998	−51.32	1	2	0.9997	−34.05
17	Avermectin B1a	20	20	0.9958	14.10	50	50	0.9911	−13.26	20	80	0.9954	−4.44	50	80	0.9904	15.66
18	Benzanilide	1	1	0.9992	−13.92	1	1	0.9985	−25.17	1	2	0.9977	−42.42	1	1	0.9983	−34.24
19	Butralin	1	1	0.9969	−11.92	1	1	0.9965	−20.67	1	5	0.9955	−47.41	1	1	0.9965	−46.02
20	Carbaryl	1	2	0.9987	−11.05	1	1	0.9943	−21.36	1	5	0.9986	−29.70	5	10	0.9947	−58.29
21	Chlormequat	1	5	0.9968	−78.31	1	5	0.9997	−87.02	2	20	0.9966	−88.99	1	5	0.9993	−91.32
22	Chlorphonium	1	1	0.9935	−8.79	1	1	0.9962	9.66	1	2	0.9974	−40.05	1	5	0.9971	−0.09
23	Cloprop	5	10	0.9989	27.46	5	10	0.9980	92.02	2	5	0.9987	73.55	5	20	0.9987	179.86
24	Cyclanilide	5	10	0.9925	−15.75	5	10	0.9992	9.95	10	50	0.9970	−33.87	5	10	0.9975	14.47
25	Cycloheximide	2	5	0.9959	15.04	2	5	0.9979	20.96	2	5	0.9993	8.64	2	5	0.9995	0.68
26	Daminozide	10	20	0.9933	−84.28	10	20	0.9938	−86.39	1	2	0.9957	−66.36	5	10	0.9909	−82.51
27	Dichlorprop	1	2	0.9988	66.40	2	2	0.9994	59.11	2	2	0.9939	146.23	1	2	0.9939	58.94
28	Diethyl Aminoethyl Hexanoate	1	1	0.9994	−36.25	1	1	0.9979	−35.66	1	1	0.9986	−42.98	1	1	0.9994	−34.75
29	Diniconazole	1	2	0.9978	4.83	1	1	0.9966	−6.73	1	2	0.9987	−18.28	1	2	0.9994	−11.45
30	Ethychlozate	1	2	0.9997	−13.03	1	2	0.9980	−16.09	2	10	0.9966	−55.70	2	5	0.9992	−17.72
31	Ethyl 1-naphthylacetate	10	20	0.9932	−40.78	10	20	0.9910	−53.81	1	5	0.9930	−29.56	1	1	0.9991	−24.44
32	Flurprimidol	1	1	0.9979	−10.89	1	1	0.9966	−2.72	1	5	0.9994	−22.08	1	1	0.9993	−1.47
33	Forchlorfenuron	1	1	0.9967	−24.88	1	1	0.9959	−40.86	1	2	0.9966	−42.65	1	2	0.9979	−33.96
34	Gibberellic	5	20	0.9995	25.57	10	20	0.9984	21.90	10	10	0.9991	18.93	2	10	0.9991	−4.54
35	Guayule	1	1	0.9992	−33.25	1	1	0.9981	−55.92	1	1	0.9995	−56.24	1	1	0.9978	−51.54
36	Inabenfide	2	5	0.9998	−3.78	1	2	0.9977	−4.75	2	10	0.9980	−11.83	5	10	0.9904	−8.35
37	Kinetin	1	1	0.9977	−39.19	1	1	0.9987	−57.46	1	1	0.9982	−58.16	1	2	0.9991	−53.98
38	Mefluidide	1	1	0.9992	21.41	1	1	0.9957	−1.20	1	2	0.9957	−13.02	1	1	0.9951	5.89
39	Mepiquat	1	2	0.9958	−37.14	1	2	0.9953	−47.88	1	2	0.9981	−56.61	1	5	0.9996	−72.30
40	Paclobutrazol	1	1	0.9993	−3.22	1	1	0.9956	−14.86	1	2	0.9986	−27.07	1	1	0.9986	−15.15
41	Phenazine-1-carboxylic acid	1	1	0.9987	−21.24	1	2	0.9965	−35.00	1	1	0.9971	−38.97	2	10	0.9989	−57.87
42	Prohexadione	5	10	0.9975	59.24	5	10	0.9983	31.32	10	20	0.9909	−16.19	1	5	0.9909	5.73
43	Prohydrojasmon	1	2	0.9958	1.57	1	5	0.9976	−11.55	1	2	0.9967	−53.24	1	5	0.9987	−25.88
44	Pyraflufen-ethyl	1	1	0.9977	−10.72	1	1	0.9964	−14.26	1	1	0.9980	−41.09	1	5	0.9987	−40.44
45	Pyribenzoxim	2	5	0.9993	−0.96	5	10	0.9985	1.02	2	5	0.9984	−10.16	2	5	0.9999	−14.72
46	Simazine	1	2	0.9983	−19.00	1	2	0.9987	−32.30	1	2	0.9995	−68.92	1	5	0.9992	−44.83
47	Thiabendazole	1	1	0.9927	161.71	1	1	0.9911	316.44	1	1	0.9932	−42.47	1	5	0.9989	−55.68
48	Thidiazuron	1	1	0.9985	−20.52	2	5	0.9990	−36.36	2	5	0.9992	−56.51	2	10	0.9969	−66.27
49	Triadimefon	1	1	0.9976	−1.16	1	2	0.9982	5.79	1	2	0.9984	−24.49	1	2	0.9990	−5.95
50	Triapenthenol	1	1	0.9989	−31.66	1	1	0.9978	−27.52	1	1	0.9993	−29.27	1	1	0.999	−18.59
51	Tribufos	1	1	0.9996	−16.48	1	1	0.9976	−17.08	1	1	0.9983	−34.17	1	5	0.9996	−80.55
52	Uniconazole	1	1	0.9989	−10.43	1	1	0.9970	−20.59	1	2	0.9979	−31.47	1	1	0.9986	−19.08
53	Zeatin	1	1	0.9941	−45.06	5	10	0.9918	−67.19	5	10	0.9921	22.33	2	10	0.9961	−52.01

**Table 5:** Results of average recoveries and the relative standard deviations (RSDs) of 10 antibiotics and 53 PGRs in four citrus fruits (*n* = 6).

No.	Compounds	Mandarin		Orange		Pomelo		Lemon	
		Recovery/%	RSD/%	Recovery/%	RSD/%	Recovery/%	RSD/%	Recovery/%	RSD/%
Antibiotics (10)									
1	4-Epi-Chlortetracycline	72.3–98.3	9.0–17.6	87.5–109.0	2.7–13.1	90.0–118.0	13.5–19.6	98.1–114.4	2.0–19.0
2	4-Epi-Oxytetracycline	78.1–97.8	5.0–5.4	75.8–89.3	6.6–8.6	79.0–109.7	3.5–5.2	85.6–98.6	3.3–10.5
3	Chlortetracycline	96.8–101.8	2.0–14.5	70.4–101.6	4.8–17.9	79.4–111.3	11.4–18.1	71.6–116.0	6.1–15.0
4	Doxycycline	71.3–90.3	2.2–12.3	72.0–89.0	5.0–10.2	79.0–100.5	4.3–19.7	71.2–89.3	12.3–17.9
5	Methacycline	70.3–104.2	3.0–5.2	70.7–87.6	5.0–15.0	71.4–85.5	6.5–10.9	72.0–116.5	10.3–17.8
6	Minocycline	66.3–76.3	14.6–14.6	71.7–90.1	4.4–17.0	72.9–95.9	9.4–18.4	72.5–76.4	7.9–8.6
7	Oleandomycin	71.1–118.6	8.7–16.0	72.7–105.1	6.3–19.4	76.6–94.9	10.2–15.6	73.6–102.2	9.5–17.9
8	Oxytetracycline	70.2–74.4	3.9–14.5	72.1–80.0	5.0–11.3	75.7–109.7	5.2–8.7	97.7–107.2	6.7–7.8
9	Sulfadimethoxine	61.8–99.8	7.8–14.2	74.0–119.6	5.7–16.0	63.3–116.7	7.0–13.9	71.5–105.4	8.7–15.6
10	Tetracycline	80.6–112.6	4.0–8.9	82.0–111.6	3.3–8.1	78.9–96.8	3.2–8.6	101.3–107.2	3.1–14.8
Plant growth regulators (53)									
1	1,3-Diphenyl urea	78.2–111.0	1.7–5.5	76.9–118.9	3.6–6.3	75.7–110.4	2.4–6.9	71.1–100.7	3.4–13.9
2	1-Naphthyl acetamide	71.2–107.6	2.2–9.0	94.9–102.8	1.4–3.6	75.5–105.8	5.4–8.5	92.9–108.7	1.2–7.0
3	2,4,5-T	100.2–118.5	3.3–8.7	104.2–112.9	1.8–4.1	89.5–110.1	2.0–7.0	92.8–106.5	1.9–7.4
4	2,4-D	102.3–118.7	1.8–12.3	100.4–112.4	2.6–6.9	101.9–118.5	2.4–5.7	90.6–105.9	2.6–4.1
5	2-Naphthyloxyacetic acid	96.5–111.0	3.3–4.8	101.9–106.1	2.6–6.0	96.9–106.0	2.2–6.7	92.1–107.6	3.9–8.3
6	2-Pyridylpropanol	92.2–103.0	2.8–4.2	70.7–96.3	1.7–4.8	70.5–98.5	1.8–2.4	93.6–102.9	2.6–13.9
7	3-Indolyl	79.4–111.4	1.7–7.9	79.8–119.2	5.0–13.0	82.4–94.8	5.2–9.8	80.2–93.7	5.6–18.0
8	4-Bromophenoxyacetic acid	97.4–118.1	1.8–6.0	104.2–105.4	1.5–4.8	75.9–111.6	1.8–15.0	98.5–107.7	2.2–5.2
9	4-Chlorophenoxyacetic acid	88.5–112.1	6.8–18.9	103.8–115.0	2.6–5.0	88.3–106.2	3.3–6.3	96.0–104.0	3.3–7.1
10	4-Fluorophenoxyacetic acid	86.9–119.2	3.8–12.8	83.6–103.7	1.6–7.1	71.6–101.5	2.6–8.0	84.0–107.8	4.3–6.6
11	4-Iodophenoxyacetic acid	105.7–117.8	2.0–5.8	102.7–107.7	4.0–4.3	109.8–117.0	3.6–8.1	84.7–99.1	1.1–5.5
12	4-Nitrophenol	89.1–119.4	3.8–11.4	78.4–116.3	2.4–9.4	91.5–106.6	2.4–3.7	97.0–108.0	2.2–14.8
13	5-Nitroguaiacolate	93.2–101.9	3.8–11.6	93.3–117.3	12.3–19.0	75.2–109.5	3.7–16.6	84.2–118.6	6.6–16.8
14	6-Benzylaminopurine	93.8–113.7	2.8–7.9	81.4–104.7	1.6–7.5	94.8–98.9	3.7–4.4	75.7–108.1	4.8–11.1
15	6-Isopentenyl aminopurine	97.4–109.6	5.9–9.7	72.7–94.9	2.7–13.2	91.3–98.2	2.8–4.2	87.3–101.4	2.8–11.5
16	Atrazine	108.5–111.4	3.2–11.1	83.6–108.7	2.8–9.7	96.1–101.8	3.6–5.6	75.3–100.5	2.9–12.5
17	Avermectin B1a	71.3–113.7	8.0–15.9	78.2–107.4	7.1–10.1	71.0–81.2	9.4–16.0	74.5–87.6	11.1–17.1
18	Benzanilide	79.4–118.6	3.2–12.4	86.2–101.0	1.6–3.7	96.1–111.5	2.1–5.9	72.3–97.9	4.1–9.1
19	Butralin	73.0–110.1	3.4–6.8	82.5–108.1	2.6–4.2	80.7–116.1	3.5–14.5	74.8–92.0	4.1–9.7
20	Carbaryl	93.5–118.2	2.9–9.9	70.3–112.6	4.3–8.2	82.8–110.6	2.5–6.7	83.7–109.8	2.9–6.6
21	Chlormequat	62.6–79.4	8.3–17.0	78.6–84.1	6.1–12.5	71.7–75.0	4.8–7.8	62.6–74.1	4.9–18.0
22	Chlorphonium	87.7–116.2	7.5–8.3	77.4–118.8	8.3–14.8	72.9–98.6	11.9–18.2	73.1–84.9	8.6–13.7
23	Cloprop	97.2–119.9	1.4–10.2	92.7–108.0	2.1–4.2	87.7–109.1	3.0–7.6	104.4–115.0	2.2–6.6
24	Cyclanilide	99.2–117.2	2.1–7.0	101.6–119.6	1.3–6.1	73.6–96.8	3.5–9.1	106.6–119.6	5.8–7.0
25	Cycloheximide	86.3–109.5	5.0–8.5	78.0–117.4	6.7–17.4	83.2–106.6	5.2–15.8	70.7–100.3	2.6–14.7
26	Daminozide	70.8–79.8	7.7–16.2	70.8–77.1	4.2–14.4	68.2–83.0	6.7–12.9	75.1–87.6	5.8–9.0
27	Dichlorprop	97.3–118.6	1.6–11.1	62.6–112.3	1.7–3.7	99.3–113.3	1.6–10.0	93.5–111.8	3.1–12.1
28	Diethyl Aminoethyl Hexanoate	106.8–111.5	3.2–5.3	85.3–104.9	1.9–3.5	74.7–107.8	2.4–3.7	71.6–106.0	4.2–9.3
29	Diniconazole	102.2–108.1	3.4–9.0	76.7–99.2	2.6–9.2	98.2–115.2	2.3–18.8	90.7–115.9	3.4–11.3
30	Ethychlozate	95.7–106.8	1.7–9.3	97.8–104.0	1.0–2.8	100.7–119.8	4.1–9.6	71.6–91.3	3.0–4.5
31	Ethyl 1-naphthylacetate	77.9–100.0	6.5–10.6	73.5–118.1	3.9–17.0	88.1–113.2	6.4–10.4	76.3–109.8	5.3–14.7
32	Flurprimidol	77.4–111.7	3.2–11.7	80.9–104.6	2.4–5.3	89.3–116.8	4.3–9.1	90.1–100.3	5.7–8.1
33	Forchlorfenuron	71.4–114.8	5.4–13.9	77.0–109.2	2.8–7.2	92.6–118.1	1.3–4.9	92.9–116.5	1.6–17.8
34	Gibberellic	75.3–107.6	6.6–14.6	79.9–106.5	3.3–12.5	99.7–109.5	5.9–13.8	73.4–95.7	4.8–16.6
35	Guayule	81.2–111.4	2.1–9.6	92.1–118.0	3.1–6.7	61.4–101.6	4.9–8.8	74.8–100.5	3.5–13.6
36	Inabenfide	87.7–113.1	2.1–9.7	83.6–119.9	2.9–5.3	102.5–116.7	3.2–6.9	99.8–111.8	2.5–4.4
37	Kinetin	73.7–94.2	0.9–6.6	70.5–89.7	3.4–5.0	63.6–94.2	2.8–5.4	70.5–74.2	2.7–7.5
38	Mefluidide	73.4–111.5	3.1–6.1	88.4–106.8	2.1–2.8	101.4–116.2	2.5–7.9	75.2–105.6	3.9–11.7
39	Mepiquat	61.4–71.2	4.7–6.3	89.5–111.4	4.3–14.4	63.1–82.5	1.9–10.9	64.6–76.0	4.3–5.2
40	Paclobutrazol	80.9–112.0	2.9–16.3	85.3–112.7	2.0–12.4	75.2–117.5	0.7–17.9	73.8–98.3	2.7–13.5
41	Phenazine-1-carboxylic acid	70.5–109.2	4.5–9.2	79.7–92.1	5.1–12.4	62.2–99.2	6.5–10.6	72.3–85.8	4.5–19.6
42	Prohexadione	78.3–118.4	2.0–10.9	81.9–115.2	2.3–7.9	81.1–111.2	3.5–14.3	80.2–99.9	3.2–10.8
43	Prohydrojasmon	96.6–104.2	14.4–16.1	92.4–109.3	9.8–15.7	63.2–99.4	8.4–15.1	70.2–106.1	11.0–14.9
44	Pyraflufen-ethyl	78.1–118.7	3.9–9.0	84.1–106.3	4.3–5.1	65.0–118.5	2.9–18.0	76.1–115.5	5.2–15.3
45	Pyribenzoxim	88.7–108.1	5.9–12.6	111.4–119.0	4.6–11.0	81.2–116.1	7.1–9.9	73.6–90.6	3.6–8.1
46	Simazine	95.1–111.2	1.6–14.1	93.4–107.9	2.6–7.4	70.3–101.3	2.9–19.9	80.1–90.3	4.7–7.4
47	Thiabendazole	102.9–118.4	2.3–3.4	97.4–114.4	1.3–1.7	99.1–116.9	1.3–3.7	72.3–92.7	1.8–4.1
48	Thidiazuron	90.1–119.5	6.5–14.3	83.6–90.0	2.8–12.2	70.3–115.1	2.4–6.5	92.0–109.0	3.8–4.3
49	Triadimefon	79.1–109.9	1.8–11.4	90.3–114.2	2.0–4.0	78.6–98.7	5.2–10.3	98.5–117.6	5.0–12.0
50	Triapenthenol	73.1–106.8	1.4–6.2	79.9–106.6	2.2–4.4	71.5–111.9	5.9–9.4	71.4–97.3	7.2–10.5
51	Tribufos	90.1–113.5	2.4–5.1	75.5–96.6	1.6–7.4	70.3–105.4	2.3–4.8	73.3–92.1	5.0–14.8
52	Uniconazole	75.9–117.6	3.0–11.8	75.8–90.6	3.4–10.5	103.4–118.7	3.0–6.7	68.8–96.7	2.0–13.2
53	Zeatin	92.2–117.8	3.8–8.3	77.9–116.6	1.0–6.5	74.8–119.3	2.5–6.6	80.7–94.9	4.6–10.0

**Table 6:** Detection of antibiotics and PGRs in four citrus fruits.

Sample	No.	Compound	Types	Detectable Amount	Detection Result	GB 2763-2021
					(mg/kg)	(mg/kg)
Mandarin	1	2-Pyridylpropanol	PGRs	3	0.004–0.027	/
	2	Cycloheximide	PGRs	1	0.036	/
	3	Paclobutrazol	PGRs	1	0.073	/
	4	Thiabendazole	PGRs	3	0.004–0.165	10
	5	Tetracycline	Antibiotics	5	0.016–0.046	/
Orange	1	2-Pyridylpropanol	PGRs	4	0.003–0.024	/
	2	Diethyl Aminoethyl Hexanoate	PGRs	1	0.002	/
	3	Paclobutrazol	PGRs	1	0.040	/
	4	Thiabendazole	PGRs	4	0.016–0.040	10
	5	Tribufos	PGRs	1	0.002	/
	6	Uniconazole	PGRs	1	0.002	0.3
	7	Tetracycline	Antibiotics	4	0.035–0.084	/
Pomelo	1	Ethyl 1-naphthylacetate	PGRs	1	0.006	/
	2	Thiabendazole	PGRs	2	0.071–0.852	10
	3	Tetracycline	Antibiotics	1	0.079	/
Lemon	1	Tetracycline	Antibiotics	2	0.059–0.120	/

/: no limit in GB 2763-2021.

## Data Availability

The original contributions presented in this study are included in the article. Further inquiries can be directed to the corresponding author.
